# Cost-Effective Laboratory Matrix Projection Micro-Lithography System

**DOI:** 10.3390/mi15010039

**Published:** 2023-12-24

**Authors:** Arslan A. Galiullin, Mikhail V. Pugachev, Aliaksandr I. Duleba, Aleksandr Yu. Kuntsevich

**Affiliations:** P.N. Lebedev Physical Institute of the Russian Academy of Science, 119991 Moscow, Russia; a.galiullin@lebedev.ru (A.A.G.); m.pugachev@lebedev.ru (M.V.P.); a.dulebo@lebedev.ru (A.I.D.)

**Keywords:** projection lithography, microfabrication, DMD matrix

## Abstract

This paper presents a home-built projection lithographer designed to transfer the image from a DLP (digital light processing) projector MEMS matrix onto the microscope objective’s field of view, where a photoresist-covered substrate is placed. The photoresist is exposed using blue light with a wavelength of 450 nm. To calibrate the device and adjust focal lengths, we utilize a red light that does not affect the photoresist. The substrate is located on a movable platform, allowing the exposure field to be shifted, enabling the exposure of designs with lateral sizes of 1 × 1 cm^2^ at a resolution of a few micrometers. Our setup showcases a 2 μ m resolution for the single frame 200 × 100 μ m^2^, and a 5 μ m resolution for 1 × 1 cm^2^ with field stitching. The exposure speed, approximately 1 mm^2^/100 s, proves to be sufficient for a variety of laboratory prototyping needs. This system offers a significant advantage due to its utilization of easily accessible and budget-friendly components, thereby enhancing its accessibility for a broader user base. The exposure speed and resolution meet the requirements for laboratory prototyping in the fields of 2D materials, quantum optics, superconducting microelectronics, microfluidics, and biology.

## 1. Introduction

Projection-mask photolithography is widely used in microelectronics for the fabrication of integrated circuits. It plays a crucial role in the process of transferring intricate patterns onto various substrates, enabling the creation of microscopic features. Most commercial lithographers are designed for substrates with diameters exceeding 2 inches. They require costly precision mechanics to ensure alignment over the substrate area with sub-micrometer precision. For laboratory prototyping in the fields of 2D materials and heterostructures [1], superconducting micro-nanoelectronics [2], quantum optics [3], microfluidics [4] and even biology [5] mask lithography is less convenient due to the requirement for creating various unique designs. Instead, laser photolithography has benefits, as it allows to pattern of custom designs with ∼1 μ m resolution. Laser lithography setups are also traditionally designed for large substrates and, therefore, are quite expensive.

Maskless projection lithography seems very promising, as it allows drawing arbitrary patterns similar to laser lithography. Projection lithography technologies, utilizing DMD (digital micro-mirror device) matrices, have changed various fields, ranging from 3D printing and sensors to microfluidics and medicine [6,7,8,9,10,11]. Generally, lithography in the laboratory should be performed on relatively tiny substrates (∼1 cm × 1 cm or even less) and does not need to be aligned over a 2–4 inch wafer size.

Most commercial lithographers are, therefore, too expensive and too huge. Laboratory technology requires a cheap and simple solution.

There are several reports of laboratory lithographers based on metallographic microscopes [12,13,14,15,16] that employ photomasks. A record small feature size for a home-build setup of 85 nm is obtained in Ref. [17]. This accomplishment was made possible by utilizing a high-aperture immersion objective (numerical aperture = 1.4) and a 365 nm-wavelength UV-LED for projection lithography using chromium photomasks. Beyond achieving maximal resolution through the optimization of the optical scheme, it is also crucial to pattern custom designs over a large area. In this paper, we introduce a home-built microscope-based projection lithographer that transfers the DMD matrix image directly into the field of view of the microobjective, where the photoresist-covered substrate is located. Thus, we can expose arbitrary designs without the need for a photomask. We expose the photoresist using a blue LED. To calibrate the device and adjust focal lengths, we exploit a red LED that does not affect the photoresist. The substrate is positioned on a movable platform, driven by stepper motors with micrometer precision. Instead of employing expensive linear stages with submicrometer steps, we have opted for a more cost-effective setup. Our setup could be employed in various laboratories for the fabrication of small devices based on flakes of 2D materials, simple metallic circuits, masks for microfluidics, and bio-compatible elements.

Several DMD-based lithographers have been consistently reported in the literature. For example, Ref. [18] discusses the enhanced precision achieved in an industrial prototype, a complicated and expensive machine. Ref. [19] reports a rather low resolution with emphasis on applications. Ref. [20] is devoted to extra-high resolution using non-linear phenomena and femtosecond laser pulses. Meanwhile, Ref. [21] shares similarities with our scheme but lacks field stitching and autofocus. In our paper, instead of concentrating on a specialized aspect, we provide a comprehensive guide on constructing a cost-effective lithographer capable of achieving a 2 μ m resolution for the single frame 200 × 100 μ m^2^ and a 5 μ m resolution for 1 × 1 cm^2^ with field stitching.

## 2. Materials and Methods

### 2.1. Construction

**Figure 1:**
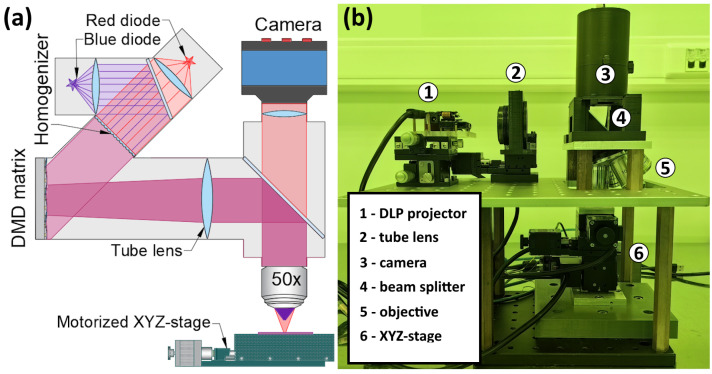
(**a**) Optical scheme for DMD-matrix based projection lithography setup. (**b**) Setup photo.

Microscopes with plano-corrected optics typically transfer the flat field of view onto the flat surface of the CCD or CMOS camera matrix. The lithographer uses the same optical scheme in the opposite direction and transfers the image of the flat DMD matrix into the microobjective field of view. A principal optical scheme and a photo of the setup are shown in Figure 1. The setup primarily consists of a custom-made metallographic microscope equipped with a series of infinity-corrected plan-achromat microobjectives (NA 0.4–0.7) mounted on a revolver nosepiece. The latter is attached to the horizontal metallic plate. Above the nosepiece there is a 45° beamsplitter. Atop the beamsplitter there is a camera with a focus lens set to infinity. In the horizontal plane, there is a tube lens and a projector. Both the tube lens and the matrix of the projector can be adjusted using mechanical micrometer-screw-driven stages.

The field of view is illuminated by either a red or a blue LED, spatially modulated by the DMD matrix (Texas Instruments, Dallas, U.S). This matrix is positioned at the focal plane of the microscope tube lens, forming its thumbnail image at the image plane of the microobjective. The tube lens projects the image of each pixel to infinity (transforming it into roughly parallel light). Subsequently, an infinity-corrected objective focuses each pixel onto the photoresist, forming a single spot of minimal size. Each pixel of the DMD matrix is an aluminum-covered square mirror with a side size of 5 μ m and a period of 5.4 μ m for our particular chip, DLP2010 (0.2″ diagonal, 854 × 480 resolution). These mirrors can switch between two positions, with one position being ON and the other being OFF. Each pixel is controlled by a pulse width modulation controller, allowing for independently adjustable doses of irradiation.

We utilize a standard module extracted from a disassembled commercial projector by iView Displays (https://www.iviewdisplays.com/) (accessed on 20 December 2023). A cheap alternative is also available at Aliexpress (https://aliexpress.com/item/1005005454618253.html) (accessed on 20 December 2023). This module comprises a light source, a DMD matrix, and a controller. The light source combines red, green, and blue LEDs (light emission diodes), allowing for an arbitrary combination (8 bits of intensity per color) for each pixel. Additionally, we connect the LED contacts to a separate controller for adjusting the brightness of the LEDs. The exposure time is controlled by displaying a topological pattern on the matrix; to halt the exposure, a black image is set on the matrix. The module features a mini-HDMI port, making its control akin to a standard display.

The magnification of the infinity-corrected microobjective (50×/20× in our case) is normally calculated assuming a 20 cm focus tube lens. However, we use a ∼10 cm focus tube lens with spherical aberration correction. As a result, we have, in fact, a 25×/10× demagnification. Specifically, each micromirror with a lateral size of 5 μ m is transformed into a 200 nm/500 nm spot at the focal plane of the objective. This spot’s geometrical size roughly corresponds to the diffraction limit of the objective, given by 1.22λ/2NA≈ 0.4 μ m/0.7 μ m, where λ=450 nm is the wavelength, and NA is the numerical aperture of the objective. In our case, NA = 0.7 for the 50× objective and NA = 0.4 for the 20× objective. The same objectives are employed to observe the projected image through the camera, facilitated by a beamsplitter. A sharp image on the camera matrix serves as an indicator of proper focusing.

In order to move the sample and adjust its focusing, we use a three-axis motorized stage that consists of two (X and Y) horizontal stages, which can be driven with 2.5 μ m steps by stepper motors. The Z-stage (by Standa (https://www.standa.lt/products/catalog/motorised_positioners?item=315&prod=motorized_vertical_translation_stages) (accessed on 20 December 2023)) has an 80 nm step, allowing us to avoid expensive piezo stages. The positions of the tube lens and the matrix module can also be fine-tuned by manipulators. We used manual manipulators and optical plates from the Aliexpress ZY Automation Store (https://aliexpress.ru/store/2782104) (accessed on 20 December 2023) as a cheap and high-quality solution.

Three optical plates, tightened by optical rods, are utilized to position all the components. The first floor hosts substrate manipulators and electronics, including stepper motor controllers, AC-DC converters, LED controllers. The second floor houses the DMD module, tube lens, and microscope objective revolving nosepiece. On the third floor, there is the beamsplitter and the camera with the objective. The components carrying the camera are 3D-printed from plastic.

### 2.2. Calibrations

We describe the major calibration procedure here. In order to adjust the focus, one should first ensure that the DMD matrix is positioned at the focal plane of the tube lens, i.e., that the images of the pixels are roughly at infinity. In this case, each pixel, after passing through the tube lens, produces a parallel beam. The manipulators are used to center the thumbnail image of central pixels within the objective’s field of view. The camera’s objective is adjusted to infinity separately. Observation of sharp elements in the camera implies that the objective is focused.

We perform auto-focus in red light to avoid undesired exposure of the photoresist. Nevertheless, due to inevitable chromatic aberrations, the focal planes in red and blue do not coincide, as illustrated in Figure 1. We therefore expose a batch (array) of identical designs varying different vertical positions of the substrate relative to the red light focus position. From the analysis of the features obtained in resist, we can determine the offset between the red and blue focuses. Once this offset is known, we perform auto-focus in red before each exposure. It is important to note that offset compensation should account for the backlash of the vertical movement (approximately ten motor steps, equivalent to 800 nm). This offset also diminishes some misalignments of the DMD matrix and tube lens focal plane, as well as addressing imperfections in the focusing of the video camera.

Similar batches are used to establish the optimal dose needed for the exposure. The dose is set by the LED exposure time. However, potentially, we may also use pulse duration modulation for each pixel, which may allow us to compensate for the nonuniform illumination of various pixel images.

### 2.3. Working with Photoresists

We use positive photoresist AZ1512HS (MicroChemicals GmbH, Ulm, Germany). The substrates (in our case, we use standard (100) Si wafers) are covered with photoresist using a spin-coater at 6000 rpm for 1 min, which corresponds to 1 μ m resist thickness. In order to demonstrate maximal resolution, we do not use any underlayers. The photoresist is baked on a hotplate at 105 °C for 1.5 min. This procedure corresponds to the Datasheet (https://www.epfl.ch/research/facilities/cmi/process/photolithography/photoresist-selection/az-1512-hs/) (accessed on 20 December 2023) and provides 1 μ m resolution. After exposure, we use the AZ 726 MIF developer for 20 s. The features in the resist are analyzed using optical and atomic force microscopes.

### 2.4. Software and Control Elements

The Arduino UNO microcontroller (Adafruit Industries, New York, NY, USA), coupled with the CNC-shield v3 (https://aliexpress.ru/item/1005005600055034.html) (accessed on 20 December 2023; ZHUANG One, Shenzhen, China), enables the control of stepper motors using A4988 drivers (Allegro MicroSystems, Manchester, NH, USA). A program has been developed for the microcontroller to communicate with the computer to execute the desired number of motor steps along each axis. Additionally, the same microcontroller is responsible for the brightness of the exposure LEDs.

The PC program connects to the DMD matrix controller, acting as a secondary monitor. It displays the image to be exposed during the lithography process. Furthermore, the program processes input from a video camera to achieve autofocus and presents the feed on the screen for real-time visual monitoring of the process.

The program incorporates the algorithms for calibrations and the precise exposure of the specified design.

### 2.5. Exposure Procedure

We begin by placing the substrate, coated with photoresist, onto the moving platform. Subsequently, we initiate an auto-focus process on the surface through the camera using a red LED employing an array of crosses. If alignment is required, the operator can identify a specific element on the substrate that has to be aligned and expose a red-colored pattern to achieve coincidence. The alignment process involves rotating the design until the desired alignment is achieved. For a single-step design, we shift the vertical stage by the previously measured number of steps to refine the focus and then perform blue light exposure. In the case of a multistep design with field stitching, we manually adjust the rotation of exposed images and the brightness of edge pixels.

## 3. Results

Three major numerical characteristics were considered in the results:

1. Lithography resolution for a single-frame exposure;
2. Resolution that allows patterning 1 cm × 1 cm square without visible field stitching problems;
3. Patterning speed at the best resolution (with and without field stitching).

### 3.1. Maximum Resolution

**Figure 2:**
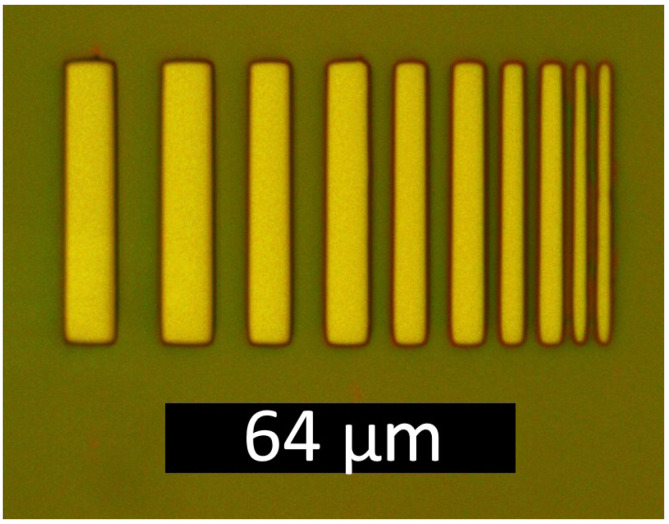
Optical microscope photograph of a developed array of stripes.

**Figure 3:**
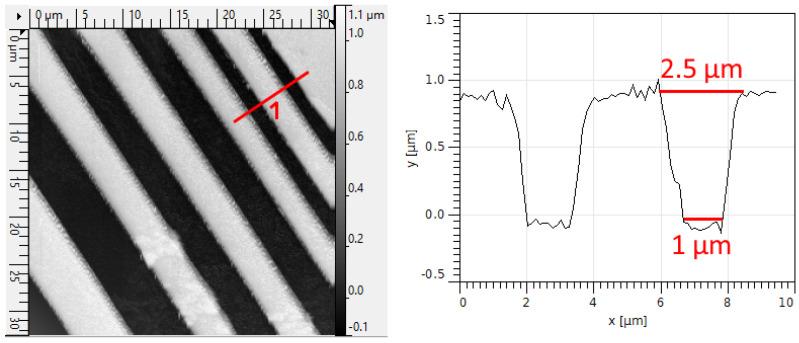
An atomic force microscope image of an array of developed stripes. The cross-sectional direction is shown in the **left panel**, and the profile along the line is displayed in the **right panel**. The dimensions of the developed part of the outer strip are indicated in the panel.

The resolution in lithography is traditionally defined as the smallest half-pitch that can be printed (pitch being the center-to-center distance of the repeated pattern). For the first parameter determination, we utilized the 50× Plan objective with NA = 0.7 (available at Aliexpress (https://aliexpress.ru/item/1005004292527909.html) (accessed on 20 December 2023) for 150 USD). Higher-aperture objectives were not employed due to the difficulty in focusing caused by our imperfect mechanics. Figure 2 and Figure 3 illustrate an example of the pattern in photoresist with ∼2 μ m resolution.

**Figure 4:**
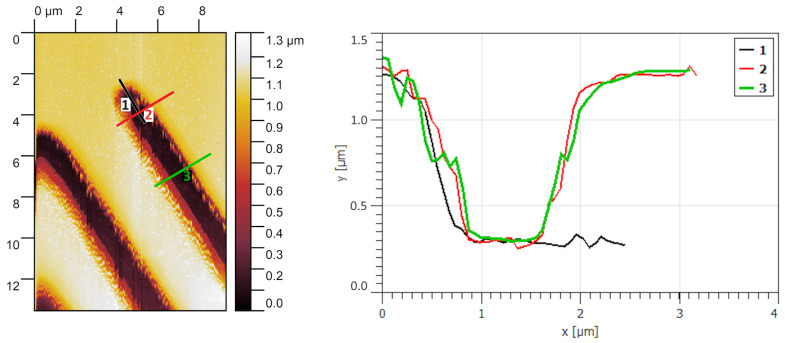
AFM image of the corner and edges of thin stripes. The **left panel** shows the colormap of the structure, and the **right** one shows the height profiles along the slices indicated in the **left panel**.

The quality of the corners and edges of the thinnest strip obtained by lithography is shown in Figure 4. The photoresist profile remains consistent across various sections of the strip, with limited squareness in the corners. The radius of curvature for the rounded corner is less than the specified resolution of 2 microns, ensuring that defects at the edges and corners do not cause the loss of resolution. If a rectangular corner shape is required, the stripes should be wider, such as the left stripes from an optical microscope image in Figure 2.

This resolution is sufficient for numerous laboratory tasks that involve working with samples ranging in size from tens of micrometers.

### 3.2. Stable Resolution with Field Stitching

**Figure 5:**
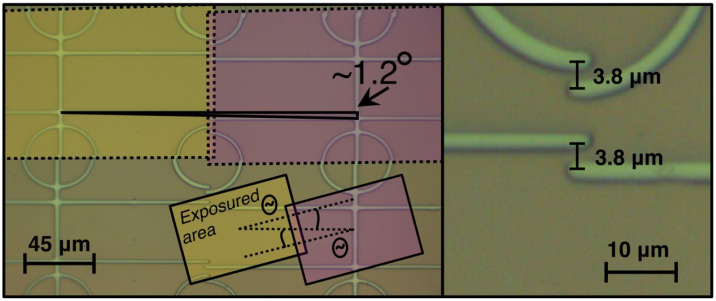
A photograph of a topological design without compensation for design rotation. The **left panel** shows a low-resolution optical image of the design. At the top of the **left panel**, the colored areas show the areas of two sequential exposures. The horizontal deviation of the exposure area from the axis of motion is about 1.2 degrees. In the bottom part of the **left panel**, the distortions are artificially enhanced for clarity. The **right panel** shows a high-resolution optical image of the bad stitching caused by non-parallelism of the matrix and incorrect X-stage movement.

The second numerical parameter, i.e., resolution for 1 cm scale lithography, is primarily constrained by imperfect field stitching. In this case, to achieve a stitched pattern without breaks, one has to make all the elements artificially thicker, i.e., decrease the resolution. Maximum stitching misalignment therefore limits the resolution. To stitch the fields, three parameters were calibrated: (i) the non-parallelism of the edges of the DMD matrix and the axes of movement of the XY-positioner; and (ii, iii) the number of steps of wafer displacement along the X and Y axes between frames. Compensation for non-parallelism was carried out by software rotating the exposure image in the editor, see Figure 5. In this figure, we present pattern stitching without non-parallelism compensation. A 3.8 μ m vertical misalignment of frames is shown in the zoom-in image (right panel). The major contribution is related to the 181 μ m $\times \sin(1.2^{\circ })$ rotational shift. Thus, the vertical/horizontal misalignment between two horizontal/vertical nearby frames is eliminated by digitally compensating for the non-parallelism between the DMD matrix’s sides and the XY axes. The number of X and Y manipulator steps was adjusted to the frame size in a separate calibration.

The discreteness of the XY stepper motor (∼2 μ m step) leads to imperfect matching for the 50× magnification objective due to the absence of lateral encoders. For a lens with a lower numerical aperture NA = 0.4 and 20× magnification (Aliexpress (https://aliexpress.ru/item/1005005982750001.html accessed on 20 December 2023), this issue was significantly less noticeable.

**Figure 6:**
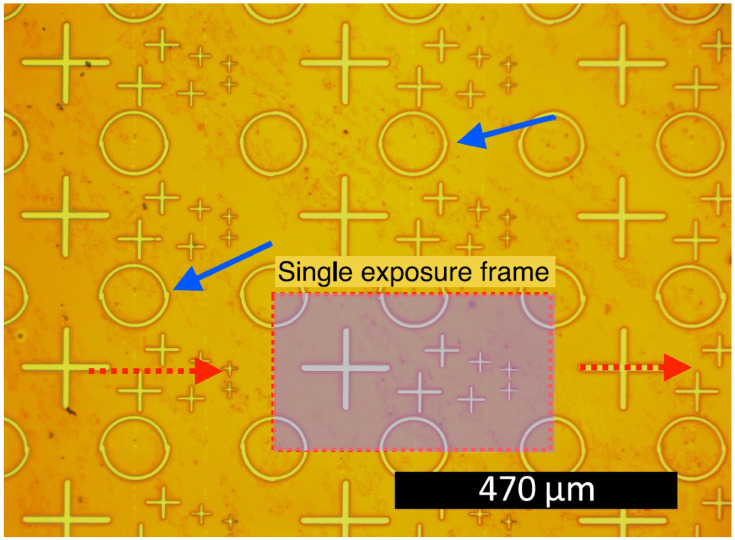
Optical microscope photograph of exposure array lithography with field stitching. The red dotted rectangle represents a single exposure frame, and the red dotted arrows indicate the direction of the field of view movement during lithography. Blue arrows highlight the similar regions where the differences in the quality of field stitching are visible.

Figure 6 illustrates an example of lithography. A periodic pattern of crosses (located at the center) and parts of circles (at the edges) was exposed. Perfect field stitching should ideally result in no distortions in the circles. However, some imperfections (indicated by the arrows) are observed.

**Figure 7:**
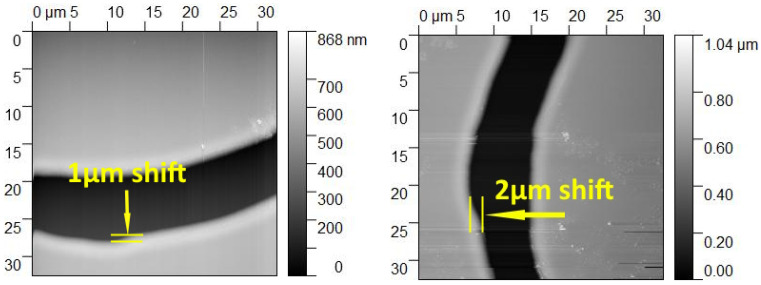
AFM images of the field stitching boundary from Figure 6.

We may evaluate the resolution of stitching as a minimal half-pitch of the design that produces continuous lines after the stitching. The major limiting factors here are the nonlinear dependence of displacement on the step number, backlash, and nonuniformity of matrix image intensity on the substrate. A typical offset of stitching area is from 1 to 5 microns, as shown in Figure 7. Correspondingly, we estimate the stitching resolution on the 1 × 1 cm^2^ substrate as 5 μ m.

Typically, only a small portion of the substrate requires high resolution, and the remaining lithography can be patterned with field stitching at a lesser resolution. Exactly this regime can be achieved in our setup.

### 3.3. Speed

The speed of patterning is limited by illumination intensity and mechanical movement. In our case, exposition of a single frame at 50× magnification (100 × 200 μ m^2^) takes 1.5 s while at 20× magnification (250 × 500 μ m^2^) it takes 4 s to expose the frame. Higher-intensity illumination could help increase the speed significantly. Indeed, the electrical power of the LED is approximately 1 W. Taking into account its ∼10% efficiency, we obtain an optical power of 100 mW. Only a small percentage of the light reaches the objective’s pupil because the tube lens turns every pixel image into a parallel beam. The ratio of the entrance pupil cross-section ( $\pi d^{2}/4$ ) to the tube lens cross-section ( $\pi D^{2}/4$ ) provides a crude estimate. This ratio, for d=3 mm and D=4 cm in our case, is equal to $d^{2}/D^{2}\approx$ 0.006. This results in 0.5 mW of optical power at the objective. The photoresist requires about 100 mJ/cm^2^ for exposure at a wavelength of 450 nm. The exposure area ( $2\times 10^{-4}$ cm^2^) requires $2\times 10^{-2}$ mJ. Therefore, we need more than 0.04 s of exposure. In fact, due to the low efficiency of the optical elements (mirrors, homogenizers, beamsplitter), this value appears to be much overestimated. Experimentally, one requires about a second to expose the resist! This means that there is considerable room for improvement on the optical side. The main advantage of the current setup is that there is no need to develop an illumination scheme; it is already included in commercial projector for both red and blue colors.

Another source of delay is the slow mechanical movement. The speed of our XY manipulator is about several hundred micrometers per second. Accordingly, for 20× and 50× objectives, drawing takes ∼1 min per mm^2^, and 0.5 mm^2^ per minute, respectively.

### 3.4. Example of Device Fabrication

**Figure 8:**
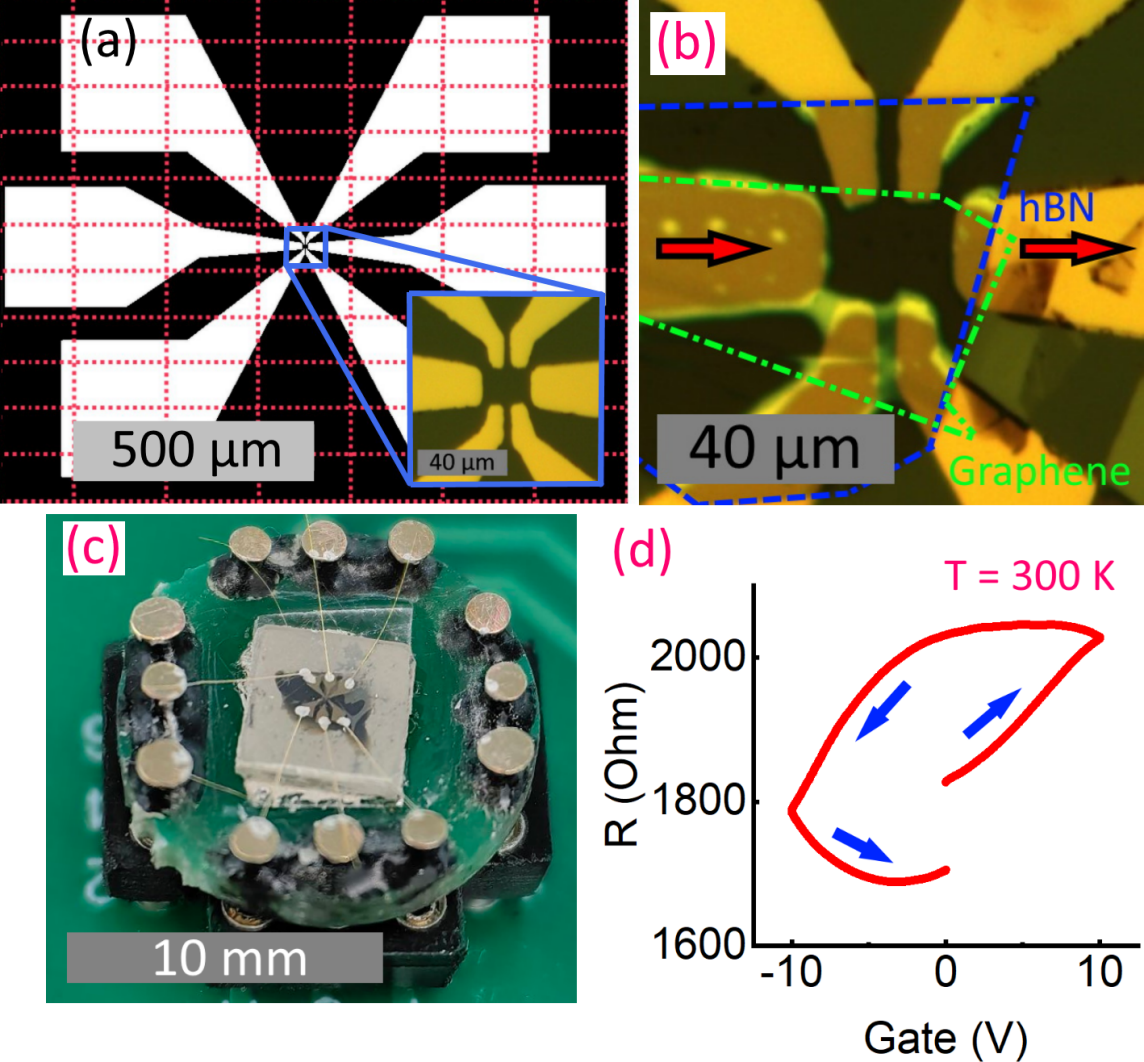
(**a**) Design layout for the bottom electrodes, used for graphene layer placement. Dotted red lines show the tiling scheme. Subfigure shows zoomed optical image of the metal electrodes before transferring graphene. (**b**) Graphene-hBN heterostructure mechanically placed on lithographically pre-patterned contacts; the arrows indicate the transport curren direction for resistance measurement; colored dotted lines indicate the boundaries of hBN and graphene flakes. (**c**) The structure mounted into the socket for measurements. (**d**) bottom-gate voltage dependence of the resistivity for the structure under consideration. Blue arrows indicate gate voltage sweep direction.

Using the presented photolithography setup, we fabricated a field-effect transistor atop the SrTiO_3_ substrate. Due to its high dielectric permittivity, a crystalline substrate of strontium titanate may operate as the bottom gate insulator for graphene devices, effectively suppressing electron-electron interactions and enabling the study of a wide range of physical phenomena [22,23,24]. In this particular case, we tested (100) SrTiO_3_ substrate from Aliexpress (https://aliexpress.ru/item/1005005973496398.html) (accessed on 20 December 2023). Contact electrodes with contact pads were first pre-patterned on a surface of SrTiO_3_ using the 50× objective. The layout and tiling are shown in Figure 8a. A 5 nm chromium/50 nm gold layer was thermally evaporated atop the developed resist, and then a lift-off was performed in acetone. The photo of the fine-resolution electrodes is shown in the inset to Figure 8a. A 5 nm Ti/50 nm Al layer was e-beam evaporated on the opposite side of the substrate. This electrode served as a back gate. Then graphene and hexagonal boron nitride flakes were exfoliated from the scotch tape to the surface of the Si wafer. The wafer was covered with 285 nm of SiO_2_ for better optical contrast. Using a homemade transfer machine [25], we assembled the heterostructure (top layer: 20 nm hBN, bottom layer: graphene) and transferred it to the electrodes on SrTiO_3_ (see Figure 8b). The sample was mounted to a carrier (socket), with silver-epoxy-bonded gold-wire contacts glued to the contact pads, shown in Figure 8c. The two-point resistivity was then measured as a function of back-gate voltage. Strong back-gate voltage dependence of the structure’s resistance at ambient temperature, shown in Figure 8d, demonstrates the functionality of both the SrTiO_3_ substrate and lithographically designed contacts. This dependence shows up anti-hysteresis, inherent to graphene on SrTiO_3_ structures [24], and elevated temperature asymmetric-in-gate-voltage signatures of the substrate recharging.

## 4. Discussion

Lithographers are highly demanded nowadays. The one presented is very cheap but rather slow and works with small substrates. The size of the exposure field in our case is approximately 100×200 μ m^2^ with a 50× objective. This field is mostly limited by the area where a flat and uniformly illuminated field is exposed. This size of this field is, in turn, limited by the objective, tube-lens, and precision of orientation of the DMD matrix with respect to the focal plane of the tube lens. Even for 2 μ m resolution in resist, the thumbnail pixel size should be less than 1 μ m, otherwise, because of addressing space discretization, inclined elements cannot be correctly exposed. Objective resolution is less crucial; nevertheless, the interference of the overlapping pixel spots in resist should be taken into account [26].

Despite having the smallest DMD matrix, the described lithographer is comparable in patterning speed to common table-top μ m resolution direct laser lithography devices. For instance, Kloe’s (https://www.kloe-france.com/en/photolithography-equipment/direct-laser-writing/direct-laser-writer-dilase-250 accessed on 20 December 2023) lithographer (KLOE, Saint-Mathieu-de-Tréviers, France) offers 10 cm/s single laser spot drawing, which implies that, if turn around time is disregarded, our standard field of view (200 × 100 μ m^2^) may be exposed in 0.2 s with 1 μ m resolution (compare with ∼1 s in our setup). A machine by Midalix (https://midalix.com/technology/ accessed on 20 December 2023) (miDALIX, Glinje, Slovenia) produces 100,000 spots per second, which is only five times higher than the total number of square micrometers in the frame but less than the entire number of pixels exposed per second.

A question arises: whether is it possible to build a bigger, faster, and higher-resolution lithographer using the same technology? Our scheme is constructed from the cheapest components and could be improved significantly. However, these improvements will dramatically increase the cost of components. We discuss them below.

1. The key element to increasing the field of exposure is the objective. On the one hand, the aperture should be rather high. For cheap objectives with NA values from 0.4 to 0.6, plan field of view is limited by several hundred μ m. A higher aperture requires more precise focusing and orientation. In our case the objective NA is not the limiting factor for the resolution. With much more expensive objectives (https://www.edmundoptics.com/p/olympus-mxplfln-20x-objective/49917/ accessed on 20 December 2023) (Olympus Corporation, Tokyo, Japan) the field of exposure should increase, and a DMD matrix with higher resolution will be needed in turn. Precise alignment between the exposed surface and the flat focal plane of the objective is essential. Additionally, these planes should be parallel to both the DMD matrix and the tube lens plane. Moreover, the microscope objective and tube lens should be coaxial and centered on the DMD matrix.
2. Illumination. In our case, the resist is exposed to non-monochromatic LED light, and we do not use apochromatic objectives to compensate for this drawback. Moreover, the light source is rather weak, so the exposure takes too much time. Replacement of the LED with an intensive light source, e.g., a pulsed laser, will solve most of the problems. However, such a replacement of the light source will require a rearrangement of the whole illumination scheme, including some lenses and a homogenizer.
3. Software could be made more sophisticated to perform alignment, corrections for autofocus, and advanced tiling of complex designs. More importantly, the pixels located at the edge of the exposure area receive different illumination from the pixels located in the depth of the exposure area. Such effects can be taken into account at the stage of image processing by digitally adjusting the brightness of the edge pixels. This procedure will improve the resolution and is especially crucial at the outer contour.
4. The next issue involves precision mechanics. The X and Y axes appear to be perpendicular with high precision and require careful control. Achieving such precision typically involves expensive mechanics. In our case, we utilize affordable stepper-driven mechanical stages and compensate for backlash by assuming it to be constant for a given mechanical stage. Since we do not require sub-micrometer positioning, we adjust the field of exposure to fit exactly the step size.

In order to build a stepper with a substrate size of at least 10 cm × 10 cm, while maintaining micrometer resolution, the requirements to the mechanical stages and field of exposure increase dramatically. Control of position with sub-micron precision becomes imperative. The device should be rather fast, and 100 μ m field of exposure becomes insufficient; requiring a total of $10^{6}$ steps for a 10 cm × 10 cm area, this would consume too much time. Furthermore, positions need to be stable. Consequently, this setup will no longer be inexpensive or simple.

In summary, the key advantages of the presented setup are:

1. Micrometer resolution: achieving a resolution of 2 μ m is demonstrated, and there is potential for even better resolution with higher aperture objectives. However, using such objectives may slow down the system and necessitate re-calibration.
2. Low cost: the total cost of components is approximately 3000 USD, making it potentially affordable for any laboratory. The breakdown includes Objective (140 USD), Tube Lens (30 USD), Motorized XYZ Stages (1700 USD), Camera (100 USD), DMD Module (500 USD), Mechanical Translation Stages (200 USD), Arduino-based Electronics (40 USD), and Optical Plates (200 USD).
3. Compact size: the table-top machine is compact and could be integrated, e.g., into a glovebox (see Ref. [27])
4. Reconfigurability. an optical breadboard-based construction allows the replacement of elements like the objective, tubelens, DMD matrix, mechanical stage, and facilitating the improvement of the setup.

## 5. Conclusions

This paper presents the realization of a home-made metallographic microscope-based lithographer-stepper. We demonstrate the attainment of a five-micrometer resolution within a 1 ${\mathrm{cm}}^{2}$ area and 2-micrometer local resolution using inexpensive optical components, the simplest DLP projector, and entry-level mechanical stages. The presented lithographer proves beneficial with individual prototypes, e.g., based on flakes of 2D materials, microfluidic elements, small metal-covered designs, e.g., traps for cold atoms, and so on. We hope our paper will inspire other laboratories to explore the development of home-made lithographers. 

## Data Availability

Data are contained within the article. No new data were created or analyzed in this study. Data sharing is not applicable to this article.
